# Matching into competitive surgical residencies: predictors of success

**DOI:** 10.1080/10872981.2023.2189558

**Published:** 2023-03-26

**Authors:** Jacob S Nasser, Anthony R Artino, Terry Kind, Xuejing Duan, Angela P Mihalic, Katherine Chretien

**Affiliations:** a School of Medicine and Health Sciences, George Washington University, Washington, DC, USA; b School of Public Health, Milken Institute, George Washington University, Washington, DC, USA; c Southwestern Medical Center, University of Texas, Dallas, TX, USA; d School of Medicine, Johns Hopkins University, Baltimore, MD, USA

**Keywords:** Residency programs, medical students, medical education, residency match, surgical specialties

## Abstract

Evidence-informed data may help students matching into competitive residency programs guide curricular activities, extracurricular activities, and residency career choices. We aimed to examine the characteristics of students applying to competitive surgical residencies and identify predictors of matching success. We identified the five lowest match rates for the surgical subspecialities listed in the 2020 National Resident Matching Program report to define a surgical residency as competitive. We analyzed a database from 115 United States medical schools regarding application data from 2017 to 2020. Multilevel logistic regression was used to determine predictors of matching. Statistical significance was set at *p* < 0.05.A total of 1,448 medical students submitted 25,549 applications. The five most competitive specialties included were plastic surgery (*N* = 172), otolaryngology (*N* = 342), neurological surgery (*N* = 163), vascular surgery (*N* = 52), orthopedic surgery (*N* = 679), and thoracic surgery (*N* = 40). We found that medical students with a geographical connection (adjusted OR, 1.65 [95% CI, 1.41 to 1.93]), and students who did an away rotation at the applied program (adjusted OR, 3.22 [95% CI, 2.75 to 3.78]) had statistically significantly increased odds of matching into a competitive surgical specialty. Furthermore, we found that students with a United States Medical Licensing Examination (USMLE) Step 1 score below 230 and Step 2 Clinical Knowledge (CK) score below 240 had increased odds of matching if they completed an away rotation at the applied program. Completing an away rotation and geographical connection to the institution may contribute more than academic criteria for selection into a competitive surgical residency after an interview. This finding may be due to less variation in academic criteria among this pool of high-performing medical students. Students with limited resources who apply to a competitive surgical specialty may be at a disadvantage given the financial burden of an away rotation.

## Introduction

Despite efforts to increase the number of medical students matching into residency positions, matching into certain surgical specialties remains competitive. Data from the National Resident Matching Program (NRMP) reveal higher proportions of unmatched U.S. senior medical students in the specialties of otolaryngology, orthopaedic surgery, plastic surgery, and neurological surgery compared to other specialties [1]. As successful matching into postgraduate training is a primary goal of undergraduate medical education, advising and supporting medical students seeking to match into the most competitive specialties can be challenging.

Various researchers have studied the influence of certain selection criteria on medical students matching into particular residency programs [2–4]. For instance, Green et al. conducted a national survey of residency program directors to identify the most influential selection criteria for residency programs and found that grades in clerkships and the USMLE Step 1 score ranked highest among the selection criteria [5]. Furthermore, researchers in particular specialties, including orthopedic surgery, plastic surgery, among others, have investigated specialty-specific factors that influence selection for residency [6,7]; however, the current literature is limited to reports of program director surveys. Moreover, there is a paucity of literature utilizing data from medical student applicants to evaluate factors that are associated with matching into competitive surgical specialties after being offered an interview.

USMLE Step 1 scores have been shown to be an integral component of the selection criteria for matching into competitive residencies [5,8–11]. Given the recent announcement by the National Board of Medical Examiners (NBME) to change score reporting for the Step 1 board examinations to pass/fail as of January 2022, identifying which other factors are important for matching into competitive specialties will potentially help students and advisors as they consider the activities that might improve students’ odds of selection into competitive specialties.

Our specific aims were to identify the characteristics of medical students applying to the most competitive surgical specialties and examine the predictors of applicants who interviewed and successfully matched compared to applicants who interviewed and did not successfully match into a competitive residency specialty. Furthermore, we conducted a supplemental analysis to determine the effect of an away rotation at the applied program and geographical connection to the program, given below average USMLE Step scores. Because this was an exploratory study, we had no a priori hypotheses regarding which factors might predict matching success.

## Materials and methods

### Database

We used data from the Texas STAR database. Data from four match cycles were used (2017 to 2020). Texas STAR was created by the University of Texas Southwestern Medical Center to help students and mentors navigate the match process more effectively and transparently [12]. Participating medical schools send their fourth-year students the Texas STAR survey on Match Day each year. In 2020, the database contained data from medical students at 115 United States medical schools. Data included self-reported board examination scores, honor society membership, research activities, publication information, volunteer experiences, among other factors, as well as which programs students applied to, interviewed at, and eventually matched [12]. The Texas STAR survey response rate averages 40% (unable to calculate for 2017, 47% in 2018, 38% in 2019, 47% in 2020). Several prior studies have utilized this database to evaluate applicant characteristics, expenditures, and success with matching into a surgical residency [13–21].

### Selection criteria

We defined a surgical specialty as competitive if it was listed in the 2020 NRMP data as one of the most competitive specialties based on match rate with the highest percentage of U.S. medical school graduates [1]. The six most competitive specialties were (1) integrated plastic surgery, (2) otolaryngology, (3) neurological surgery, (4) thoracic surgery, (5) vascular surgery, and (6) orthopedic surgery. Students in the Texas STAR database were included if they participated in the survey from 2017 to 2020.

### Outcome and predictor variables

Our primary outcome variable was matching into one of the six competitive specialties. Predictor variables included Alpha Omega Alpha honor status (AOA status), USMLE Step 1 score, USMLE Step 2 Clinical Knowledge (CK) score, other academic degrees, away rotation status (i.e., whether an away rotation was conducted at the program to which they applied), geographical connection, couples match status, number of honored clerkships (clerkship for which an A/Honors was awarded), honored specialty applying for (whether or not student received A/Honors for clerkship of specialty applied to), number of research experiences, number of peer-reviewed publications, number of abstracts/posters/presentations, number of leadership roles, and the number years off for research. Geographical connection was defined using the survey responses of students and may indicate the applicant has a hometown in the geographical region, attended college or research fellowship in the region, or conducted a visiting rotation in the region. The questions included on the Texas STAR survey are included in Appendix.

### Statistical analysis

We calculated descriptive statistics for all variables and then conducted univariate analyses to examine differences between students matching into competitive specialties and students not matching into competitive specialties. We then calculated a multilevel logistic regression model to identify predictors of matching into a program. The multilevel logistic regression used data from each individual application, rather than data on an individual applicant. As such, each student applicant could be included multiple times in the regression analyses based on the number of programs they applied to. Multilevel modelling is commonly used in such situations, where there are multiple observations per participant (applications nested within individuals).

Categorical variables were analyzed using a chi-square test and continuous variables were analyzed using a Kruskal Wallis test. Statistical significance level was set at *p* < 0.05. Finally, we conducted a supplemental analysis using multilevel logistic regression to identify if completing an away rotation at the program or having a geographical connection to an institution increased odds of matching given above or below average USMLE Step 1 and Step 2 CK scores. This study was deemed exempt by the Institutional Review Board at the George Washington University School of Medicine and Health Sciences.

## Results

### Basic student characteristics

Our sample included a total of 1,448 students who submitted 25,549 applications from 2017 to 2020. All students included in our analysis applied to a single specialty. A total of seven students were not offered an interview after applying. Basic student characteristics for those who applied to one or more of the six most competitive surgical specialties are listed in Table 1. On average, each student applied to 71 programs and interviewed at 18 programs. The majority of students in our sample (94%) had a Step 1 score greater than or equal to 230. Additionally, most students (92%) had a Step 2 score greater than or equal to 240. Of the students who applied to one or more of the six most competitive surgical specialties, less than half (43%) reported AOA status. Furthermore, we found that the average number of honored clerkships for students applying to one or more of the six most competitive surgical specialties was 4.4 (SD ±2.3). Although few students in the study had no peer-reviewed publications (*N* = 69), all of them had some type of research experience.

**Table 1.** Student Characteristics.

N (%)	
*Academic Predictors*	
**USMLE Step 1 Score**	
190–209	7 (0.5)
210–219	18 (1.2)
220–229	68 (4.7)
230–239	191 (13.2)
240–249	437 (30.2)
250–259	523 (36.1)
≥260	204 (14.1)
**USMLE Step 2 CK Score**	
210–219	8 (0.6)
220–229	22 (1.5)
230–239	86 (5.9)
240–249	252 (17.4)
250–259	531 (36.7)
≥260	543 (37.5)
Unknown	6 (0.4)
**Honored Clerkships** (Mean ± SD)	4.44 (2.31)
**AOA Status**	
Yes	620 (42.8)
No	763 (52.7)
Absent School Chapter	65 (4.5)
**Other Degrees**	
PhD	29 (2.0)
MS	131 (9.0)
MPH	38 (2.6)
Other	75 (5.2)
None	1,175 (81.1)
**Honored Specialty Applying For**	
Yes	1,042 (72.0)
No	85 (5.9)
Unknown	321 (22.2)
*Research Predictors*	
**Research Experiences** (Mean ± SD)	5.5 (2.9)
No Research Experiences	0 (0)
**Peer-Reviewed Publications** (Mean ± SD)	4.0 (3.5)
No Peer-Reviewed Publications	69 (4.8)
**Abstracts/Posters/Presentations** (Mean ± SD)	6.7 (3.7)
No Abstracts/Posters/Presentations	6 (0.4)
**Research Year**	
Yes	196 (13.5)
No	1,184 (81.8)
Unknown	68 (4.7)
*Geographic Predictors*	
**Geographic Connection**	
Yes	249 (17.2)
No	1,199 (82.8)
*Other Predictors*	
**Number of Leadership Roles** (Mean ± SD)	4.3 (±2.8)
No Leadership Roles	95 (6.6)
**Away Rotation Status**	
Yes	66 (4.6)
No	1,382 (95.4)
**Couples Match Status**	
Yes	100 (6.9)
No	931 (64.3)
Unknown	417 (28.8)

### Predictors of successful matching

Academic and research variables were not associated with significantly increased odds of successfully matching into a particular program (Table 2). Students with a geographical connection to the program were just over one and a half times more likely to match compared to students without a geographical connection (adjusted OR, 1.65 [95% CI, 1.41 to 1.93], *p* < 0.001). Additionally, medical students who elected to do an away rotation at the program were 3.22 times more likely to match into a program compared to students who did not do an away rotation (adjusted OR, 3.22 [95% CI, 2.75 to 3.78], *p* < 0.001).

**Table 2.** Predictors of matching into top surgical residency specialties^π^.

	OR (95% CI)	*P-Value*
*Academic Predictors*		
**USMLE Step 1 Score**		
190–209	Reference	
210–219	0.90 (0.17, 4.73)	0.900
220–229	0.73 (0.16, 3.45)	0.694
230–239	0.87 (0.19, 4.01)	0.857
240–249	0.83 (0.18, 3.82)	0.814
250–259	0.86 (0.19, 3.93)	0.843
≥260	0.83 (0.18, 3.87)	0.816
**USMLE Step 2 CK Score**		
210–219	Reference	—
220–229	0.99 (0.23, 4.27)	0.990
230–239	0.94 (0.25, 3.59)	0.931
240–249	0.98 (0.27, 3.57)	0.975
250–259	0.96 (0.26, 3.49)	0.951
≥260	0.94 (0.26, 3.40)	0.920
**Honored Clerkships** (Mean ± SD)	1.00 (0.96, 1.04)	0.908
**AOA Status**		
No	Reference	—
Absent School Chapter	0.98 (0.67, 1.43)	0.900
Yes	0.88 (0.74, 1.05)	0.154
**Other Degrees**		
None	Reference	—
PhD	1.20 (0.76, 1.89)	0.443
MS	0.93 (0.73, 1.20)	0.589
MPH	0.83 (0.53, 1.30)	0.419
Other	1.03 (0.72, 1.48)	0.853
**Honored Specialty Applying For**		
No	Reference	—
Yes	1.01 (0.73, 1.38)	0.967
*Research Predictors*		
**Research Experiences**	1.00 (0.97, 1.02)	0.798
**Peer-Reviewed Publications**	0.99 (0.97, 1.02)	0.477
**Abstracts/Posters/Presentations**	1.00 (0.97, 1.02)	0.851
**Research Year**		
No	Reference	—
Yes	1.03 (0.82, 1.29)	0.796
*Geographic Predictors*		
**Geographic Connection**		
No	Reference	—
Yes	1.65 (1.41, 1.93)	<0.001
*Other Predictors*		
**Number of Leadership Roles**	1.00 (0.98, 1.03)	0.902
**Away Rotation Status**		
No	Reference	—
Yes	3.22 (2.75, 3.78)	<0.001
**Couples Match Status**		
No	Reference	—
Yes	1.00 (0.80, 1.25)	0.988

*Notes*: ^π^ Variables in this multivariable include Step 1 and Step 2 USMLE scores, number of honored clerkships, AOA status, degree type, honored specialty applying for, research experiences, presence of a geographical connection, number of leadership roles, and away rotation status.

### Away rotation status and geographical connection on matching

Students with a USMLE Step 1 score<230 and a USMLE Step 2 CK score<240 had a statistically significant increased odds of matching if they conducted an away rotation at the specific program (OR, 5.61 [95% CI, 3.17 to 9.92], *p* < 0.001). Additionally, students with Step 1 scores ≥ 230 and Step 2 CK scores ≥ 240 had increased odds of matching if they did an away rotation at the specific program (Table 3). Students with a geographical connection were at increased odds of matching; however, this result was only statistically significant for students with Step 1 scores ≥ 230 or Step 2 CK scores ≥ 240.

**Table 3.** Influence of away rotation and geographical connection on matching given USMLE step score.

	Unadjusted OR (95% CI)	*P-Value*
*USMLE Step 1 < 230 and USMLE Step 2 CK < 240*		
**Away Rotation**		
No	Reference	—
Yes	5.61 (3.17, 9.92)	<0.001
**Geographical Connection**		
No	Reference	—
Yes	1.30 (0.72, 2.36)	0.383
*USMLE Step 1 ≥ 230 and USMLE Step 2 CK ≥ 240*		
**Away Rotation**		
No	Reference	—
Yes	4.71 (4.24, 5.24)	<0.001
**Geographical Connection**		
No	Reference	—
Yes	2.02 (1.83, 2.22)	<0.001

### Match rate by specialty and year

**Figure 1.**
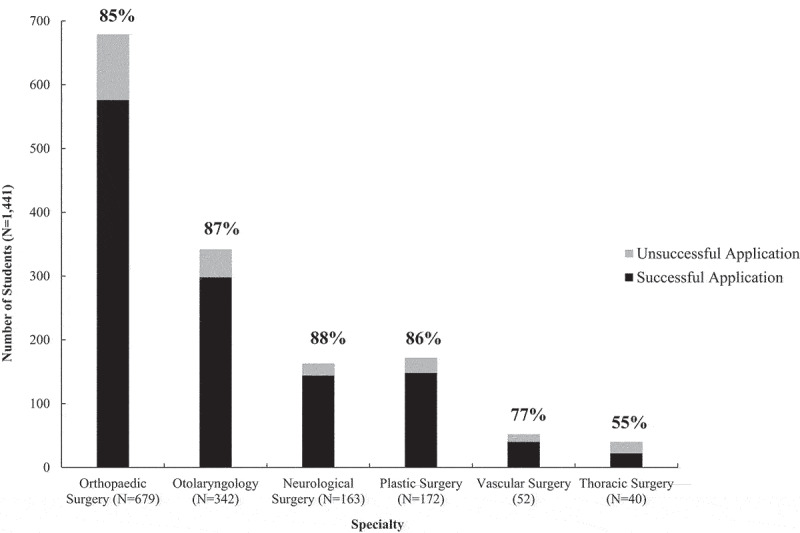
Match rate after interview stratified by Specialty^α,χ^.

Figure 1 represents the percentage of applications who had a successful match, stratified by specialty. Of the specialties analyzed in our study, most students interviewed at orthopaedic surgery (47%) or otolaryngology (24%) programs. Among the specialties included in our study, students interviewing at a neurological surgery program had the highest match rate after interviewing (88%), followed by otolaryngology (87%). On the other hand, students interviewing at a thoracic surgery program (55%) had the lowest match rate after interviewing. Furthermore, when comparing the rate of matching after interview for each year included in the study, the match rate did not differ significantly from year to year. Table 4 includes match rates for different specialties over the study period.

**Table 4.** Match rate by year and specialty.

	*2017*				*2018*				*2019*				*2020*			
	N	Matched	Unmatched	% Matched	N	Matched	Unmatched	% Matched	N	Matched	Unmatched	% Matched	N	Matched	Unmatched	% Matched
Neurological Surgery	4	4	0	100	38	37	1	97	54	48	6	89	67	55	12	82
Orthopedic Surgery	30	20	10	67	176	144	32	82	210	179	31	85	263	233	30	89
Otolaryngology	13	13	0	100	81	75	6	93	105	86	19	82	143	124	19	87
Plastic Surgery	14	12	2	86	41	38	3	93	42	37	5	88	75	61	14	81
Thoracic Surgery					6	4	2	67	20	11	9	56	14	7	7	50
Vascular Surgery	3	3	0	100	11	9	2	82	15	12	3	80	23	16	7	70

αIncludes all students offered an interview; seven students were not offered an interview after applying. χThe number above the bars in the figure represent the match rate after interview.

## Discussion

Providing medical students with evidence-informed information to guide their curricular and extracurricular activities is needed, as matching into competitive surgical residency programs continues to become more difficult. Additionally, such information can help guide residency career choices. In our study, we utilized the Texas STAR database to identify the factors related to successful matching for students applying to the six most competitive surgical specialties. Although the majority of students included in our study had higher-than-average USMLE Step 1 and 2 scores, numerous research experiences, among other favorable selection criteria, these variables were not associated with an increased (odds) of matching into one of the most competitive surgical specialties. Additionally, we found that students with below average USMLE scores had increased odds of matching if they performed an away rotation at the program. In addition, an away rotation was more helpful for students with below average USMLE Step scores, as the odds ratio was higher for students with below average scores compared to above average scores.

The NRMP’s biennial Charting Outcomes of the Match research report is published to provide transparency to medical students regarding criteria for matching. The general characteristics of students applying to these competitive subspeciality programs are similar to those of the 2020 Charting Outcomes in the Match. This indicates that our sample may be generalizable to most allopathic medical students applying into these specialties, not just those at the institutions included in our sample. Individualized data on each application sent to every program that a student applied, which is included in Texas STAR, permits for further analysis of the factors influencing residency selection.

Various researchers have examined the selection criteria that contribute to successful matching into residency programs [3,7,10,22]. The Texas STAR database was used by Kremer et al. to examine the factors that increased the odds for matching into any medical specialty. Unlike that study, our study goal was to identify factors specific to students applying to competitive surgical specialties, since these criteria may differ. Additionally, we wanted to identify if geographical connections and away rotations could help students with below average USMLE Step scores. Kremer and colleagues examined data from 10,000 students included in the database to identify the odds of being offered an interview according to various academic, research, and extracurricular factors. The authors identified AOA status, Gold Humanism Honor Society, away rotation at a program, and geographical connection as increasing odds for an interview offer. In our study, we also found that geographical connection and away rotation status at a program increased odds of matching into a competitive surgical specialty. Findings from other studies on surgical subspecialties also align with some of our findings about specific samples of medical students applying to surgical subspecialties [23–25]. Specifically, USMLE scores, research experiences, AOA status, graduation from a top medical school, and having a surgical mentor were discussed as factors contributing to increased odds of matching.

Recent literature reinforces the importance of geographical location and away rotations on successful matching [22,26,27]. A study conducted by Gauer et al. found that matching into certain residency programs was significantly associated with USMLE Step 1 and USMLE Step 2 scores [8]. Indeed, the sample examined in our study was comprised of students who mostly had high USMLE Step scores. As such, range restriction was a factor and may partly explain why USMLE Step scores did not emerge as an important distinguishing factor in our sample of high-achieving medical students. In other words, students applying to the surgical specialties included in our study typically apply with competitive board scores, as this is integral to higher match success; thus, mentors and advisors likely encourage students to consider their USMLE Step performance when counseling them on applications. Nonetheless, we found that conducting an away rotation at a program was statistically significantly associated with increased odds of matching, despite USMLE scores. Higher USMLE Step scores, better academic performance, and AOA status are, intuitively, factors that would increase the odds of an individual matching into a particular specialty as they are indications of outstanding academic performance. For competitive surgical specialties, we suspect there is less variation in these criteria. Most students applying to the specialties included in this study have multiple research experiences and above average USMLE Step scores. Therefore, more subjective measures, including experiences and performance on an away rotation, potential networking on an away rotation, letters of recommendation, and interview performance may weigh more heavily for this specific population. Although we cannot evaluate the qualitative aspects of the selection process, including interview and personal statements, this is an avenue of future research.

Away rotations may provide medical students a better understanding of their desired specialty, and these rotations give students the opportunity to establish relationships with mentors who may aid in their success in a particular field. Fereydooni and colleagues examined medical students’ motivations for pursuing a career in integrated vascular surgery and the logistics of away rotations. The authors found that 83% of students matching into an integrated vascular surgery residency completed an away rotation. This study highlighted the importance of away rotations in permitting students an opportunity to assess the factors that foster their choice to pursue residency at a particular program [28]. Additionally, Baldwin et al. retrospectively reviewed medical student application data for orthopaedic surgery and found that the number of away rotations performed by a student increased odds of a match success [27]. Additionally, an away rotation may allow for the development of a meaningful relationship with a mentor, that may lead to a letter of recommendation or involvement in research, which may further increase an applicant’s chances to match into a particular program.

Costs associated with residency applications and away rotations in surgical subspecialities have been investigated in the literature and evidence shows that the costs for applying to certain surgical subspecialties is higher than other subspecialties [13,16,20,29,30]. Specifically, Gordon et al. used the Texas STAR database to compare the costs of students applying to competitive surgical residencies, and the authors found application costs to vary depending on subspeciality and geographic region [20]. Tese findings highlights the need to develop strategies to reduce variation in the costs of residency applications and limit costs overall. Away rotations may represent substantial financial burden for students applying to certain specialties, presenting an opportunity to develop strategies to combat the high costs associated with residency applications. For example, research indicates that some students may pay as much as $2,500 to perform away rotations [28]. Given the potential advantages of away rotations, as highlighted in the current study, it is important to ensure that these experiences are available to all students, regardless of their financial status or resources available at their home institution. For example, strategies may be adopted by host and welcoming institutions to provide low-resource students the opportunity to attend an away rotation or reap similar benefits. Such strategies may include stipends, food allowances, complementary housing, among other methods [26,28,31].

In addition, the COVID-19 pandemic led to the development of virtual electives for students who could not rotate in-person. Best practices for virtual experiences could be shared to maximize their benefits for learners. Furthermore, some students do not perform well in a standardized testing environment or have structural barriers to testing performance, such as less access to test preparatory materials, and an away rotation may present them with the opportunity to showcase their knowledge in a practical way. Thus, institutions who require a particular score before even offering an invitation to do an away rotation should consider assessing applicants in a more holistic manner. Finally, the development of a national policy limiting the number of away rotations that students can perform could promote equity, as well as address overall costs.

Geographical connections to a particular program or region may communicate meaningful information to a residency committee in terms of likelihood of a student’s sincere interest in the program. Such data may be communicated through permanent address, school address, personal statements, interview, or through leadership and volunteer experiences. Recently, the Electronic Residency Application Service (ERAS) introduced geographic preference capabilities within their supplemental application during 2021–2022 and 2022–2023 residency application cycles [32]. Additional insights from their data will be helpful in understanding the importance of communication around applicant geographic preferences.

Our study has limitations. The Texas STAR database contains self-reported fourth-year student data that is completed on Match Day or later each year, exposing the dataset to potential recall bias. Nonetheless, students have access to their application data after submission. However, the mean student board scores in our study match the NRMP’s Charting Outcomes in the Match scores, likely indicating a representative sample. Importantly, this database does not contain data regarding interviews, letters of recommendation, class ranking, personal statements, and other factors that may predict successful matching. Future research might consider collecting data from program directors on interview performance and the quality of letters of recommendation at particular programs to determine the influence of interviews. Nonetheless, we included data from over 1,448 students and 115 medical schools (out of a total of 155 total accredited MD-granting institutions in 2019) [33]. Finally, because these data come from a cross-sectional survey, we cannot make causal inferences regarding the relationships observed in this study. We acknowledge that successful matching and match rates for specialties are influenced by many factors, including number of positions available, interview performance, and other factors not included in this study. Numerous metrics can be used to define a surgical specialty as competitive, including size of the applicant cohort compared to the number of positions available and number of ranks per applicant needed to match in a given specialty. Nonetheless, we decided to utilize the match rates listed in the NRMP report to define a surgical residency as competitive. Future studies may focus on identifying which metric is most indicative of competitiveness. Additionally, the match algorithm is driven by applicant preference and this influences where a student ultimately matches. Although the Texas STAR database includes data from students in numerous medical schools, the absence of data from all accredited MD schools may influence differences in match rates observed in our study and NRMP’s Charting Outcomes data. Multilevel logistic regression did not control for specific specialty, and this may influence the results as certain specialties vary in regards to the value of specific applicant characteristics during ranking.

## Conclusion

In this analysis, examining predictors of matching into competitive surgical specialties, we found that away rotations completed at the program to which a student applies to and geographical connections are integral components of the selection process. Away rotations provide medical students with immense opportunities to network, get involved with specialty-specific research, and familiarize themselves with the specialty, among other benefits. Given the financial implications associated with away rotations, institutions across the country should work to ensure all students have access to these benefits, regardless of socioeconomic status or their ability to perform an away rotation. Future work should aim to gather more comprehensive evidence regarding the residency selection process to provide further transparency for students as they prepare their residency applications to programs across the country. Additionally, these studies may identify trends in the applications submitted by students to determine the effect of various medical education policies on the residency application process.

## Highlights

1. Students with geographical connection to an institution or students who performed an away rotation at an institution are more likely to match into competitive surgical specialties.
2. Students with below average board scores had increased odds of matching into a competitive surgical specialty if they completed an away rotation at the applied program.
3. Away rotations and geographical connections to an institution may contribute more than academic criteria for selection into a competitive surgical residency after an interview, as academic criteria may not be distinguishing in this self-selected applicant pool.

## Data Avalibaility Statement

The Texas STAR program is committed to keeping these data confidential to ensure the privacy of the individual applicants. Therefore, we did not provided these data upon submission.

## Appendix.

**Table:**

*Academic Predictors*	
USMLE Step 1 Score	Please select the range in which your USMLE Step 1 score falls.
USMLE Step 2 CK Score	Please select the range in which your USMLE Step 2 CK score falls.
Honored Clerkships	On how many core clerkships did you receive a final grade of A/Honors?
AOA Status	Are you a member of Alpha Omega Alpha Honor Medical Society?
Other Degrees	Please indicate any additional degree you will have completed at the time of your graduation from medical school.
Honored Specialty Applying For	Did you receive a final grade of A/Honors on your home rotation in this specialty?
*Research Predictors*	
Research Experiences	Please indicate the number of research experiences listed on your residency application.
Peer-Reviewed Publications	Please indicate the number of peer-reviewed publications listed on your residency application that have been submitted, accepted, or published.
Abstracts/Posters/Presentations	Please indicate the number of abstracts, posters, and presentations listed on your residency application.
Research Year	Please indicate if you took an additional year (or more) for the purpose of research.
*Geographic Predictors*	
Geographic Connection	Indicate if you had a geographic connection to the area.
*Other Predictors*	
Number of Leadership Roles	Please indicate the number of leadership positions listed on your residency application.
Away Rotation Completed at Institution	Indicate if you completed an away rotation at the program.
Couples Match Status	Did you participate in the Couples Match?

*Adapted from the Texas STAR Database Survey.

## References

[1] National Resident Matching Program (NRMP). Main residency match. 2021. Avaliable from: https://mk0nrmp3oyqui6wqfm.kinstacdn.com/wp-content/uploads/2021/05/MRM-Results_and-Data_2021.pdf. cited August 10, 2021.

[2] Chen JY, Heller MT. How competitive is the match for radiology residency? Present view and historical perspective. J Am College Radiol. 2014;11(5):501–11. 10.1016/j.jacr.2013.11.011 24793041

[3] Mitsouras K, Dong F, Safaoui MN, et al. Student academic performance factors affecting matching into first-choice residency and competitive specialties. BMC Med Educ. 2019;19(1):241. 31262294 10.1186/s12909-019-1669-9 PMC6604174

[4] Curtin LS, Signer MM. Ensuring the integrity of the national resident matching program. JAMA. 2017;318(23):2289–2290. 29114782 10.1001/jama.2017.16269

[5] Green M, Jones P, Thomas JJ. Selection criteria for residency: results of a national program directors survey. Acad Med. 2009;84(3):362–367. 19240447 10.1097/ACM.0b013e3181970c6b

[6] Bernstein AD, Jazrawi LM, Elbeshbeshy B, et al. An analysis of orthopaedic residency selection criteria. Bull Hosp Jt Dis. 2003;61(1/2):49–57. 12828380

[7] LaGrasso JR, Kennedy DA, Hoehn JG, et al. Selection criteria for the integrated model of plastic surgery residency. Plast Reconstr Surg. 2008;121(3):121e–125e. 10.1097/01.prs.0000299456.96822.1b 18317094

[8] Gauer JL, Jackson JB. The association of USMLE step 1 and step 2 CK scores with residency match specialty and location. Med Educ Online. 2017;22(1):1358579. 28762297 10.1080/10872981.2017.1358579 PMC5653932

[9] Claiborne JR, Crantford JC, Swett KR, et al. The plastic surgery match: predicting success and improving the process. Ann Plast Surg. 2013;70(6):698–703. 23673567 10.1097/SAP.0b013e31828587d3

[10] Weissbart SJ, Stock JA, Wein AJ. Program directors’ criteria for selection into urology residency. Urology. 2015;85(4):731–736. 25817098 10.1016/j.urology.2014.12.041

[11] Super N, Tieman J, Boucher K, et al. Recent trends in applicants and the matching process for the integrated plastic surgery match. Ann Plast Surg. 2013;71(4):406–409. 23407248 10.1097/SAP.0b013e31824ca654

[12] UT Southwestern Medical Center. Texas STAR. 2021. Avaliable from: https://www.utsouthwestern.edu/education/medical-school/about-the-school/student-affairs/texas-star.html. Accessed August 6, 2021.

[13] Benjamin WJ, Lenze NR, Farlow JL, et al. Cost of the otolaryngology residency application process: comparison with other specialties and equity implications. OTO open. 2022;6(3):2473974X221119150. 10.1177/2473974X221119150 PMC9382083 35990815

[14] Lenze NR, Mihalic AP, DeMason CE, et al. Predictors of otolaryngology applicant success using the Texas STAR database. Laryngoscope Investig Otolaryngol. 2021;6(2):188–194. DOI:10.1002/lio2.549 PMC8035942 33869750

[15] Lenze NR, Mihalic AP, Kovatch KJ, et al. Impact of the COVID-19 pandemic on the 2021 otolaryngology residency match: analysis of the Texas STAR database. Laryngoscope. 2022;132(6):1177–1183. 34515992 10.1002/lary.29860 PMC8661614

[16] Gordon AM, Sarac BA, Drolet BC, et al. Total costs of applying to integrated plastic surgery: geographic considerations, projections, and future implications. Plast Reconstr Surg Glob Open. 2021;9(12):e4058. 34963875 10.1097/GOX.0000000000004058 PMC8694517

[17] Gordon AM, Malik AT, Scharschmidt TJ, et al. Cost analysis of medical students applying to orthopaedic surgery residency: implications for the 2020 to 2021 application cycle during COVID-19. JBJS Open Access. 2021;6(1). DOI:10.2106/JBJS.OA.20.00158 PMC8352616 34386683

[18] Gordon AM, Malik AT. Costs of US allopathic medical students applying to neurosurgery residency: geographic considerations and implications for the 2020–2021 application cycle. World Neurosurg. 2021;150:e783–789. 33831614 10.1016/j.wneu.2021.03.149

[19] Gordon AM, Conway CA, Sheth BK, et al. How did coronavirus-19 impact the expenses for medical students applying to an orthopaedic surgery residency in 2020 to 2021? Clin Orthop Relat Res. 2022;480(3):443–451. DOI:10.1097/CORR.0000000000002042 34913886 PMC8846343

[20] Gordon AM, Ahlering TE. How does geographic region affect the total and individual costs for medical students applying to the competitive surgical residencies? J Surg Educ. 2022;79(1):147–156. 34535435 10.1016/j.jsurg.2021.08.016

[21] Christensen BR, Becnel CM, Chan LP, et al. A comparison of match outcomes between traditional medical degree and dual-degree applicants. PLoS ONE. 2020;15(12):e0244147. 33338062 10.1371/journal.pone.0244147 PMC7748145

[22] Kremer TR, Kremer MJ, Kremer KP, et al. Predictors of getting a residency interview: differences by medical specialty. Med Educ. 2020;55(2):198–212. 32750181 10.1111/medu.14303

[23] Vaysburg DM, Cortez AR, Hanseman DJ, et al. An analysis of applicant competitiveness to general surgery, surgical subspecialties, and integrated programs. Surgery. 2021;170(4):1087–1092. DOI:10.1016/j.surg.2021.03.035 33879334

[24] Meyer AM, Henderson A, McDonald CE, et al. Factors associated with matching into surgical specialties. J Surg Res. 2022;270:300–312. 34731727 10.1016/j.jss.2021.09.020

[25] Rinard JR, Garol BD, Shenoy AB, et al. Successfully matching into surgical specialties: an analysis of national resident matching program data. J Grad Med Educ. 2010;2(3):316–321. 21976075 10.4300/JGME-D-09-00020.1 PMC2951766

[26] Villwock JA, Hamill CS, Ryan JT, et al. The role of the away rotation in otolaryngology residency. Otolaryngol Head Neck Surg. 2017;156(6):1104–1107. 28349746 10.1177/0194599817698431

[27] Baldwin K, Weidner Z, Ahn J, et al. Are away rotations critical for a successful match in orthopaedic surgery? Clin Orthop Relat Res. 2009;467(12):3340–3345. 19582529 10.1007/s11999-009-0920-9 PMC2772936

[28] Fereydooni A, Ramirez JL, Morrow KL, et al. Factors influencing medical student choices in the integrated vascular surgery match: implications for future post-pandemic residency matches. J Vascular Surg. 2021;74(4):1354–1361.e4. 10.1016/j.jvs.2021.05.014 34023431

[29] Agarwal N, Choi PA, Okonkwo DO, et al. Financial burden associated with the residency match in neurological surgery. J Neurosurg. 2017;126(1):184–190. 27058197 10.3171/2015.12.JNS15488

[30] Camp CL, Sousa PL, Hanssen AD, et al. The cost of getting into orthopedic residency: analysis of applicant demographics, expenditures, and the value of away rotations. J Surg Educ. 2016;73(5):886–891. DOI:10.1016/j.jsurg.2016.04.003 27184179

[31] Margolin EJ, Gordon RJ, Anderson CB, et al. Reimagining the away rotation: a 4-week virtual subinternship in urology. J Surg Educ. 2021;78(5):1563–1573. 33483279 10.1016/j.jsurg.2021.01.008

[32] Association of American Medical Colleges (AAMC). About the supplemental ERAS application. 2021. https://students-residents.aamc.org/applying-residencies-eras/about-supplemental-eras-application. Accessed July 19, 2021.

[33] Association of American Medical Colleges (AAMC). AAMC medical school enrollment survey: 2019 results. Avaliable from: https://www.aamc.org/media/47726/download. Accessed August 6, 2021.

